# Eccentric Exercise Program Design: A Periodization Model for Rehabilitation Applications

**DOI:** 10.3389/fphys.2017.00112

**Published:** 2017-02-23

**Authors:** Michael O. Harris-Love, Bryant A. Seamon, Tomas I. Gonzales, Haniel J. Hernandez, Donte Pennington, Brian M. Hoover

**Affiliations:** ^1^Muscle Morphology, Mechanics and Performance Laboratory, Clinical Research Center—Human Performance Research Unit, Veterans Affairs Medical CenterWashington, DC, USA; ^2^Geriatrics and Extended Care Service/Research Service, Veterans Affairs Medical CenterWashington, DC, USA; ^3^Department of Exercise and Nutritional Sciences, Milken Institute School of Public Health, The George Washington UniversityWashington, DC, USA; ^4^Physical Medicine and Rehabilitation Service, Veterans Affairs Medical CenterWashington, DC, USA; ^5^Department of Physiology and Biophysics, College of Medicine, Howard UniversityWashington, DC, USA

**Keywords:** eccentric exercise, periodization, rehabilitation, physical therapy, isokinetic exercise, muscle performance, muscle strength

## Abstract

The applied use of eccentric muscle actions for physical rehabilitation may utilize the framework of periodization. This approach may facilitate the safe introduction of eccentric exercise and appropriate management of the workload progression. The purpose of this data-driven Hypothesis and Theory paper is to present a periodization model for isokinetic eccentric strengthening of older adults in an outpatient rehabilitation setting. Exemplar and group data are used to describe the initial eccentric exercise prescription, structured familiarization procedures, workload progression algorithm, and feasibility of the exercise regimen. Twenty-four men (61.8 ± 6.3 years of age) completed a 12-week isokinetic eccentric strengthening regimen involving the knee extensors. Feasibility and safety of the regimen was evaluated using serial visual analog scale (VAS, 0–10) values for self-reported pain, and examining changes in the magnitude of mean eccentric power as a function of movement velocity. Motor learning associated with the familiarization sessions was characterized through torque-time curve analysis. Total work was analyzed to identify relative training plateaus or diminished exercise capacity during the progressive phase of the macrocycle. Variability in the mean repetition interval decreased from 68 to 12% during the familiarization phase of the macrocycle. The mean VAS values were 2.9 ± 2.7 at the start of the regimen and 2.6 ± 2.9 following 12 weeks of eccentric strength training. During the progressive phase of the macrocycle, exercise workload increased from 70% of the estimated eccentric peak torque to 141% and total work increased by 185% during this training phase. The slope of the total work performed across the progressive phase of the macrocycle ranged from −5.5 to 29.6, with the lowest slope values occurring during microcycles 8 and 11. Also, mean power generation increased by 25% when eccentric isokinetic velocity increased from 60 to 90° s^−1^ while maintaining the same workload target. The periodization model used in this study for eccentric exercise familiarization and workload progression was feasible and safe to implement within an outpatient rehabilitation setting. Cyclic implementation of higher eccentric movement velocities, and the addition of active recovery periods, are featured in the proposed theoretical periodization model for isokinetic eccentric strengthening.

## Introduction

The use of eccentric muscle actions for the purpose of therapeutic exercise has gained greater acceptance in light of the growing evidence that positive adaptations can result without incurring excessive muscle damage (Lindstedt et al., 2001; LaStayo et al., 2007; Lindstedt, 2016). Older adults and individuals with chronic conditions often have limited exercise tolerance and may require specialized exercise programming provided by rehabilitation professionals. The ability to elicit muscle and neural adaptations to exercise in people with compromised metabolic and cardiovascular capacity makes eccentric exercise an intriguing training option. Rehabilitation interventions featuring eccentric exercise appear to have similar efficacy and safety to concentric training for the management of conditions such as coronary artery disease, musculoskeletal conditions such as tendinopathies and knee osteoarthritis (OA), as well as chronic neurodegenerative diseases such as Parkinson disease (Gur et al., 2002; Dibble et al., 2006; Roig et al., 2008; Gluchowski et al., 2015). Moreover, there is some evidence to suggest that the gradual introduction and progression of eccentric training loads result in large strength gains in older adults without incurring adverse changes in serum creatine kinase, tumor necrosis factor-α, or other clinical markers of muscle damage (LaStayo et al., 2007). While eccentric training has been shown to be an effective form of therapeutic exercise, at risk populations such as older adults may be more susceptible to muscle injury or impaired recovery in response to a bout of high force muscle actions (Lovering and Brooks, 2014; Gluchowski et al., 2015). These competing risks and benefits highlight the need to codify principles of eccentric exercise program design in order to promote the safe and effective implementation of this strengthening method.

Program design for strength training involves the organization of exercise volume and intensity for the purpose of attaining a specific performance goal. Among the most frequently used approaches to program design is periodization (Lorenz et al., 2010; Fleck, 2011). Periodization is a general method of dividing a training regimen into discrete phases marked by systematic loading and recovery phases. These training phases have been traditionally structured in accordance to an annual calendar or the timing of competitive sporting events, and are further defined by periods of exercise specificity and skill acquisition (Stone et al., 1981; Rhea and Alderman, 2004; Miranda et al., 2011). All forms of program design involving progressive resistance exercise (PRE) require an initial exercise prescription. Professional organizations and scientific societies such as the National Strength and Conditioning Association (NSCA) and the American College of Sports Medicine (ACSM) provide guidance on establishing an appropriate exercise prescription (Haff, 2012; American College of Sports Medicine, 2014). Core elements of the exercise prescription include workload assignment, exercise frequency and duration, workload progression, and exercise mode. The broad goals of the exercise prescription for strength training are identifying an appropriate workload and volume to promote safe exercise participation, improving musculoskeletal heath and general fitness, and preventing the onset and severity of chronic disease and geriatric syndromes (American College of Sports Medicine, 2014). The exercise prescription for strength training certainly shares many of the elements of program design. Nevertheless, the components of the exercise prescription concerning the safe assignment of workload and selection of exercise mode rise in importance when introducing older adults or those with physical limitations to a formalized exercise routine.

It has been noted that rehabilitation interventions involving strength training are often absent of clear guidelines on specific training variables and rarely incorporate periodization into the exercise program (Lorenz et al., 2010). Noting the need for rehabilitation programs to better integrate formal elements of exercise program design, Hoover et al. (2016) have stated that, “Periodization principles should be an integral part of sport physical therapy training and lexicon.” This observation is even more pronounced when considering the lack of formal program designs for eccentric training (Murtaugh and Ihm, 2013). While the roles of eccentric muscle actions in biomechanics and strength training have been studied for decades (Dudley et al., 1991; Lindstedt, 2016), the exercise prescription and exercise programming specific to this type of muscle action have been less explored. These elements of the eccentric PRE regimen have important implications for both athletic training and general rehabilitation. This Hypothesis and Theory paper is a data-driven approach to examine the feasibility and safety of the applied use of eccentric muscle actions for the purpose of physical rehabilitation. The objectives of this study are to consider an approach to the initial eccentric exercise prescription in outpatient rehabilitation settings, and propose an eccentric exercise periodization model featuring a decision algorithm for workload adjustments when using accommodating resistance devices. In addition, data from older exercise participants are used to examine the potential impact of the eccentric power-velocity relationship on training variables within a periodization model involving isokinetic exercise.

## Materials and methods

### Participants

This longitudinal pilot study was conducted to determine the feasibility of a periodized eccentric strength training program involving older adults with musculoskeletal impairments. Thirty-eight people were successfully screened for inclusion into the study and 25 people enrolled with one person failing to complete the study due to conflicting time commitments. Therefore, 24 community-dwelling veterans completed participation in the study at the Washington DC Veterans Affairs Medical Center (DC VAMC) Clinical Research Center. All of the participants were older men (age: mean = 61.8 years, *SD* = 6.3 years; height: mean = 178.6 cm, *SD* = 8.4 cm; weight: mean = 102.7 kg, *SD* = 15.0 kg; BMI: mean = 32.3, *SD* = 5.1) with knee osteoarthritis confirmed by physician assessment and radiological report (Kellgren–Lawrence grade: median = 3, interquartile range = 2–3). The sample included 23 African-American participants and 1 Caucasian participant. All enrolled participants reported that they were not receiving physical therapy treatment or participating in a formal exercise program, and they were categorized as being untrained (no regular bouts of exercise for at least 30 min in duration, with a frequency ≥3 times per week, over a period of ≥3 consecutive months). The study was approved by the DC VAMC Research and Development Institutional Review Board and registered with Clinicaltrials.gov (NCT02098096). Signed informed consent was obtained from all study participants prior to data collection.

### Procedures

The eccentric strength training and peak torque assessments were completed using an isokinetic dynamometer (Biodex System 4, Biodex Medical Systems, Shirley, NY) as previously described (Hernandez et al., 2015). Isokinetic data for peak torque, total work, and mean power were obtained at a sample rate of 100 Hz using the Biodex System 4 Advantage software. Torque-time curves were reviewed to check for movement artifacts and to ensure that recorded force values were obtained at the specified angular velocity. Gravity correction was calculated prior to testing and exercise sessions with the participant's knee extended in the terminal testing position while the relaxed limb was fastened to the attachment pad. Gravity correction factors varying >10% from the baseline value were recalculated until consistent measurements were obtained within acceptable limits. The cushion deceleration parameter was maintained at the lowest setting to ensure that maximal movement time would be spent at the specified angular velocity. System checks and calibration procedures were performed per the manufacturer's guidelines. The primary data collection activities for this study are broadly categorized as strength testing and eccentric strength training procedures.

### Strength testing

All participants were tested in a seated position in the dynamometer chair. The dynamometer chair was adjusted for proper seat height, backrest angle and position, and chair position relative to the powerhead location, height, and angle of orientation. Participant positioning and dynamometer chair adjustments were used to attain 90° of hip flexion and knee flexion prior to testing, with the lateral femoral condyle aligned with the dynamometer shaft axis of rotation. Positioning was attained and checked using the measurement guides on the dynamometer chair, powerhead, and base, along with palpation of bony landmarks at the knee joint, and inclinometer measures of joint position. The dynamometer attachment for knee extension/flexion strength testing was used during the conduct of the tests and the attachment pad height was adjusted for each participant to be ~3 cm proximal to the calcaneus. This positioning approach for the attachment pad avoids potentially painful or distracting contact of the apparatus with the calcaneus during terminal knee flexion. The participants were stabilized in the dynamometer chair to prevent compensatory motions and promote reproducible testing sessions. The stabilization straps of the dynamometer chair were fastened at the shoulders, pelvis, and ipsilateral thigh. The knee attachment stabilization strap was fastened to the ipsilateral lower leg at the level of the attachment pad. All participant and dynamometer positions were recorded in order to replicate the testing conditions during subsequent testing and exercise visits.

Muscle strength was assessed via isokinetic dynamometry at 60 and 180° s^−1^ using methods from previously published protocols (Pincivero et al., 1997; Harris-Love, 2005). Reciprocal isokinetic knee extension and flexion testing was conducted within a range of motion (ROM) of ~90°–100°, depending on participant tolerance and the available passive ROM. These ROM limits, also ensured that terminal extension did not exceed 10° in order to protect the knee joint at the end ROM of the dynamometer excursion. Participants completed five maximal repetitions at the selected angular velocity with the tested limb tested in a random order. Approximately 1 min of rest was provided between the five-repetition testing bouts. Participants were allowed to stabilize their trunk with their hands on the dynamometer handles, but were instructed to not attempt to pull their trunk forward during testing. Visual feedback was provided to the participant from the torque-time curve depicted on the dynamometer computer screen, and verbal cuing was provided as needed concerning attempted compensatory motions during testing. Warm up activity involving four to six repetitions of submaximal isokinetic knee extension and flexion at 180° s^−1^ was performed with each limb before engaging in maximum volitional repetitions. A familiarization session was provided to each participant prior to strength testing to orient each person to isokinetic testing and obtain the dynamometer chair positioning settings. This session also provided the opportunity to determine if strength testing would result in any lower extremity pain or discomfort given the clinical population involved in the study. The peak torque was derived from the mean value of the highest three peak torque values from the five-repetition test. Participants were instructed to perform the testing movement as forcefully and rapidly as possible while avoiding the Valsalva maneuver. Similar approaches to strength assessment have been found to be reliable by other investigators (intraclass correlation coefficients, ICCs, exceeding 0.92 with an estimated measurement error of 8%) in younger and older adults (Pincivero et al., 1997; Hartmann et al., 2009). Also, the investigators' laboratory reliability is acceptable for isokinetic knee extension/flexion strength testing in the 60 and 180° s^−1^ conditions as conducted in this study. Intraclass correlation coefficients (ICC_2, 1_) range from 0.97 to 0.99 (*df* = 30; *p* < 0.001, with lower bound 95% confidence intervals that range between 0.95 and 0.99) and a standard error of the measurement up to 21.0 N m, in a cohort of older African American adults. The same two investigators conducted all of the strength assessment and eccentric strength training sessions.

### Eccentric strength training

The eccentric PRE program for the knee extensors and flexors was 12 weeks in duration with two scheduled training bouts per week, for a total of 24 training sessions as previously reported (Harris-Love, 2005; Hernandez et al., 2015). At least 1 day of rest was required between training sessions. Participant positioning and warm up activities prior to the exercise bouts were identical to the procedures used during the strength assessment sessions for this study. The Biodex System 4 dynamometer settings for the exercise sessions were also similar to the settings used for strength assessment with the notable exception of the operation mode. The reactive eccentric exercise mode was used for reciprocal knee extension and flexion. This mode of isokinetic exercise requires the participant to exert at least 10% of the assigned torque limit (~22.5–44.5 N m above the targeted workload in this study) in order to engage the mechanized motion of the dynamometer powerhead shaft. The concentric peak torque values were used to calculate the estimated isokinetic eccentric peak torque for the initial workload assignment (Hernandez et al., 2015). This approach was taken as a precaution given the arthritic conditions within the older men featured in our sample who were untrained and naïve to the eccentric muscle action exercise stimulus:

(1) $$\begin{array}{l} \tau_{\text{ecc}}=\left(\tau_{\text{con}}\right)\left(1.35\right) \end{array}$$

where τ = torque obtained at 60 or 180° s^−1^, ecc = eccentric, and con = concentric.

In summarizing the periodization approach used in this study, the entire 12-week regimen constituted the initial “macrocycle.” The macrocycle was designed to introduce the exercise stimulus to individuals naïve to eccentric training and advance their program to include workloads sufficient to optimally induce skeletal muscle adaptations. “Mesocycles” typify extended phases of specific training that may last a few weeks to ~2 months. In this exercise program, the first mesocycle constituted an introductory period of eccentric training and included the familiarization and acclimatization phases of the macrocycle (i.e., the first 3 weeks of training). The second mesocycle included the progression phase in its entirety as the workload and movement velocity were systematically increased until the end of the regimen. “Microcycles” are brief training periods that may last 3–7 days, and were used in this program to represent two non-consecutive exercise sessions within a 1-week period (Lorenz et al., 2010). A phased introduction to the exercise stimulus and manipulation of program variables such as workload, volume, and repetition speed (e.g., angular velocity) provides a systematic approach to minimizing the risks and maximizing the benefits associated with eccentric strength training.

**Figure 1:**
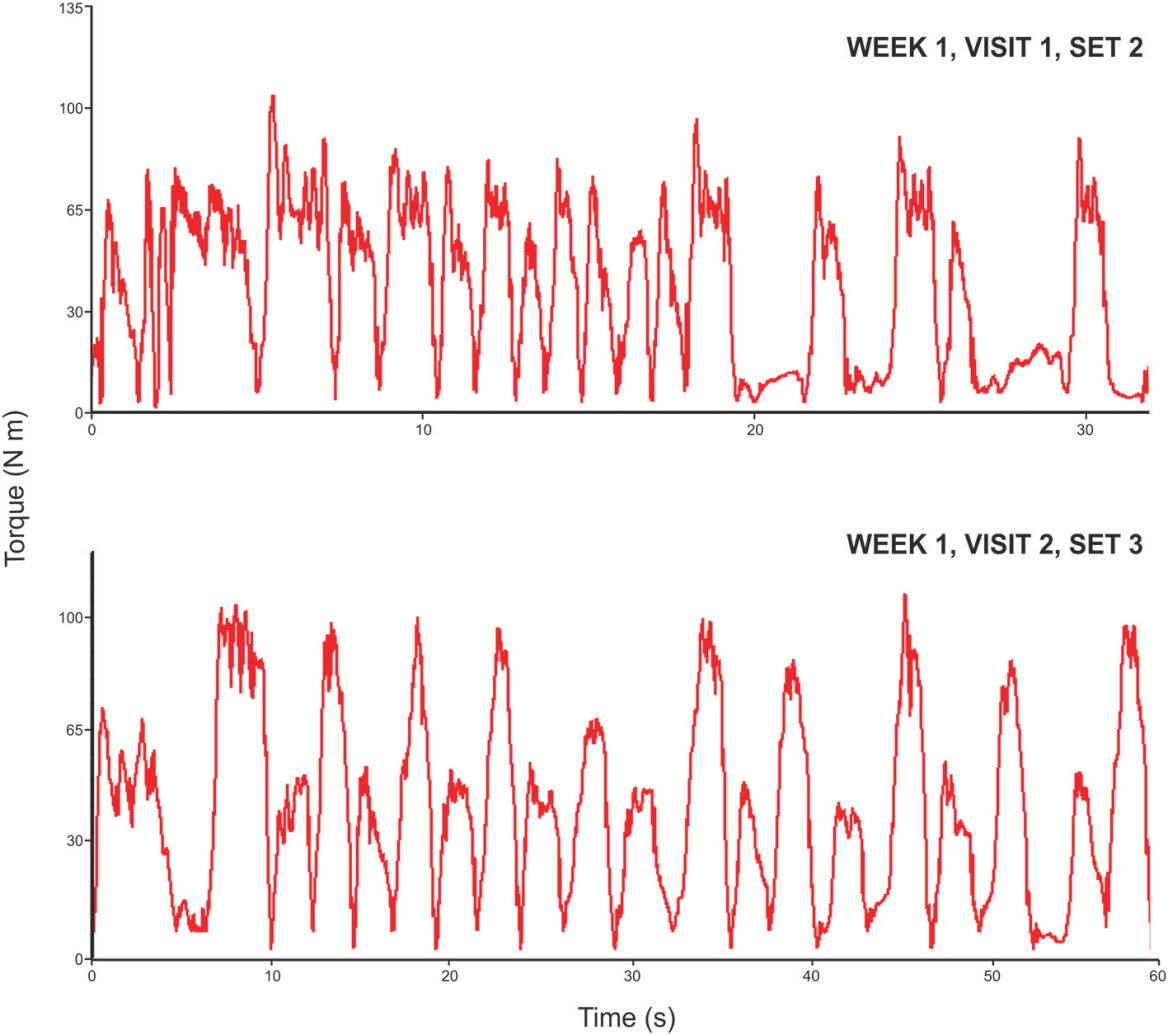
**Eccentric torque-time curves during the familiarization phase of the strength training program**. The exemplar data shows the transition in the isokinetic eccentric motor performance (in the 45°/s exercise condition) observed during the familiarization phase of the strength training program. The torque-time curve for knee extension and flexion observed during session one exhibits a high degree of variability based on the eccentric knee extension mean repetition interval time (coefficient of variation, CV = 68%). However, this variability decreases by the end of the familiarization phase during session two (CV = 12%).

The initial eccentric training phases of the periodized eccentric training program included a 1-week *familiarization phase* to allow the participants to experience the muscle recruitment patterns associated with isokinetic eccentric exercise, followed by a 2-week *acclimatization phase* to induce the muscle action history-dependent protective response to subsequent eccentric muscle actions under similar or progressively higher workloads. Selected data was obtained from the familiarization phase to reflect the motor learning that occurs over the initial exercise sessions. Previous investigators have noted that basic temporal data may reflect the pattern of torque curves (Watkins and Harris, 1983). Consequently, the mean repetition interval (sec) was measured from the peak torque value of each knee extension repetition and also expressed as a coefficient of variation (CV) value (Figure 1).

**Figure 2:**
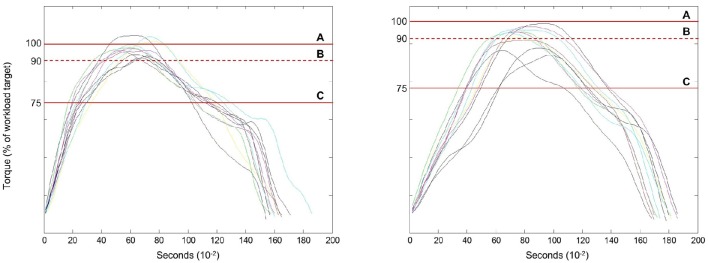
**Workload adjustments based on the eccentric torque-time curves relative to the visual torque targets**. Strengthening exercise involving the use of accommodating resistance requires an assessment of intra-session anaerobic fatigue data to make appropriate workload adjustments. The line graphs depict exemplar torque-time curves for eccentric knee extensor exercise. The torque targets include: Line A–the designated exercise workload, Line B–this dashed line is the lower-bound ±10% tolerance level for the acceptable attainment of peak torque during each repetition, and Line C–the indicator of peak torque levels that fall 25% below the designated exercise workload. The left panel captures a successful exercise set that would result in a 5% increase in the designated workload, whereas, the right panel reveals additional markers of anaerobic fatigue, but not fatigue levels that reach the criterion limit of 25%. The exercise performance shown on the right panel would result in the maintenance of the designated workload for the subsequent exercise session.

Following these early phases of training to become oriented to isokinetic eccentric strengthening, the participants began the *progression phase*. Exercise during microcycle 4 through microcycle 12 was centered on inducing training adaptations using incremental adjustments in exercise workload. The safe adjustment of the target workload was aided by the use of a progression algorithm that allows for workload modification following each exercise session. The patterns of the torque-time curves are monitored in real-time during the exercise bouts to detect excessive declines in torque in any of the exercise sets. Torque-time curves with an observed decline >25% of the target workload (for >2 consecutive repetitions following verbal cueing) denotes significant fatigue based on *a priori* decision criteria. This magnitude of intra-session fatigue resulted in the workload goal being decreased by ~5% for the next exercise session. Similar declines of the torque-time curves that were >10%, but <25%, of the target workload resulted in the retention of a stable workload goal in the subsequent exercise session. Lastly, completion of the all sets meeting the target workload with an absent or minimal torque decline (<10%) resulted in a 5% increase in the target workload during the subsequent exercise session (Figure 2). The eccentric training regimen featured 3 sets of 10 repetitions from microcycle 1 to microcycle 5. The exercise volume was then progressed from 3 sets to 4 sets from microcycle 6 to microcycle 12, with the movement speed transitioning from 60 to 90° s^−1^ between microcycle 7 and microcycle 9. The late phase training sessions from microcycle 10 to microcycle 12 included all exercise sets at 90° s^−1^. Pilot work within the laboratory suggested that eccentric angular velocities faster than 90° s^−1^ would have been difficult to complete for untrained clinical populations who were also unfamiliar with the mode of exercise.

The selection of the initial limb to be exercised was random, and the completion of each set was proceeded by 1 min of recovery time. Visual and verbal feedback was provided in a similar manner as the strength assessment sessions, and also included verbal cuing to address exercise technique issues, if needed. In addition, verbal cueing was used to determine if a participant could attain the visual workload targets if diminished torque-time curves were observed in real time. Visual analog scale (VAS, 0–10) values for self-reported musculoskeletal pain of the lower extremity were documented prior to each exercise session (Gallasch and Alexandre, 2007). Moreover, verbal communication between the participant and the tester was used to determine the presence of any discomfort during the exercise session or upon its completion. Following each exercise session, any instance excessive fatigue based on the torque-time curves was noted, the updated workload targets were documented in the exercise log, and total work was recorded. In addition, knee extensor peak torque and mean power attained during the eighth microcycle were analyzed since the exercise volume was evenly divided between two sets each at the 60–90° s^−1^ condition with the same visual torque targets at both angular velocity settings. Data concerning the eccentric power-velocity relationship obtained during exercise may provide insight about how to best manipulate the isokinetic angular velocity as an element of the periodization program. Taken together, the review of the VAS values, basic exercise adherence data, and mean power-velocity data help to inform the feasibility of the periodized eccentric strengthening regimen. The initial macrocycle and general workload progression scheme used in this study is provided in Table 1.

**Table 1:** **Initial Eccentric Training Macrocycle**.

**MACROCYCLE–INITIAL**											
***Familiarization***	***Acclimatization***		***Progression***								
**Mesocycle1**			**Mesocycle 2**								
**Micro 1**	**Micro 2**	**Micro 3**	**Micro 4**	**Micro 5**	**Micro 6**	**Micro 7**	**Micro 8**	**Micro 9**	**Micro 10**	**Micro 11**	**Micro 12**
**Low target workload**			**Initial training workload**	**Progressive increase in target workload and power generation**							
45° s^−1^, 3 × 10	60° s^−1^, 3 × 10	60° s^−1^, 3 × 10	60° s^−1^, 3 × 10	60° s^−1^, 3 × 10	60° s^−1^, 4 × 10	60° s^−1^, 3 × 1; 90° s^−1^, 1 × 10	60° s^−1^, 2 × 1; 90° s^−1^, 2 × 10	60° s^−1^, 1 × 1; 90° s^−1^, 3 × 10	90° s^−1^, 4 × 10	90° s^−1^, 4 × 10	90° s^−1^, 4 × 10
**TARGET ECCENTRIC WORKLOAD (% PEAK TORQUE) AND PROGRESSION**											
40–50%	50%	60%	70%	±5%	±5%	±5%	±5%	±5%	±5%	±5%	±5%
**ATTAINED ECCENTRIC WORKLOAD (% PEAK TORQUE)**											
40%	50%	60%	70%	75%	85%	95%	105%	113%	125%	132%	141%

*Micro, microcycle; deg, degrees; s, second. Baseline estimated eccentric peak torque based on concentric peak torque at 60° s^−1^ and a conversion factor of 1.35; the workload adjustment algorithm was applied after each session from microcycle 5 to microcycle 12*.

*The primary features of the periodized eccentric strength training program include introductory training phases to allow for motor learning related to eccentric isokinetic exercise and submaximal, incremental exposure of the training stimulus in order to induce the repeated bout effect. The second mesocycle incorporates the progression phase of the exercise regimen for the systematic increase in workload and movement velocity*.

### Data analysis

Descriptive statistics were used to convey participant characteristics, and exemplar and aggregate data regarding exercise performance, VAS values, and exercise adherence. Parametric data are expressed as means and standard deviations (*SD*), and non-parametric data are shown as median values with the interquartile range. The participants' motor performance exhibited during the eccentric isokinetic exercise was examined from exemplar data and conveyed as the variability (i.e., coefficient of variation, CV) of the knee extensor mean repetition interval times. The change in the total work values observed during the progression phase of the eccentric exercise program was also evaluated. These delta values were generated in the 60° s^−1^ condition and used to compare individual participant performance, and facilitate the visual depiction of the data. Slope analysis of the total work values was assessed across two consecutive microcycles (e.g., a total of four exercise sessions), in a serial fashion, within the progressive phase of the eccentric training program in order to identify an increase, decrease, or plateau in exercise capacity. The analysis of consecutive microcycles has value since markers of eccentric muscle damage, including residual delayed onset soreness, could have a precipitating event followed by observed deleterious effects that overlap across 2 training weeks (Lavender and Nosaka, 2007; Kanda et al., 2013).

Inferential statistics were used to evaluate velocity-dependent muscle performance outcomes. Eccentric torque-velocity and power-velocity relationships were derived from the torque and power values attained from two intra-session eccentric exercise sets at 60 and 90° s^−1^. Consequently, paired *t*-tests were used for the analysis of difference concerning the torque and power values across each testing condition (Portney and Watkins, 2009). Peak torque and mean power data were further characterized by scaling the values to the 60° s^−1^ testing condition and then visually depicting the proportional change in values in the 90° s^−1^ testing condition using a radar graph. Analyses from the dominant limb data are presented given that the general statistical findings in this report were not dependent on limb dominance (as determined by the participant limb preference to kick a ball based on self-report). Statistical analyses were performed using PASW Statistics for Windows, Version 18.0 (SPSS Inc., Chicago, IL, USA). The α level was set at 0.05, and two-tailed *p* < 0.05 were considered significant for all inferential statistics.

## Results

The supervised eccentric exercise program was well-tolerated by the study participants and the study was conducted without incurring an intervention-related serious adverse event. One non-study related adverse event was reported due to the death of a patient several months following the intervention period. However, 29% of those successfully screened for inclusion into the study opted to not participate. Completion of the regimen required the completion of 24 exercise visits and 3 assessment visits over a 12-week period. The reasons provided by enrolled subjects for their non-participation were “scheduling issues,” “job demands,” and anticipated “difficulty with appointments.” Regarding those that chose to participate in the study, their mean VAS values for musculoskeletal pain were 2.9 ± 2.7 at the start of the regimen and 2.6 ± 2.9 upon completion of eccentric strength training program. Also, during the progressive phase of the macrocycle, exercise workload increased from 70% of the estimated eccentric peak torque to 141% and total work increased by 185% during this training phase.

Regarding muscle performance measures, the findings for the knee flexors were similar to the knee extensors. Therefore, the analysis of the data for the knee extensors is provided to characterize the periodized eccentric strengthening program and feasibility of the regimen. The exemplar data shown in Figure 1 reflects the transition in the motor performance observed during the familiarization phase of the eccentric isokinetic exercise program. Intra-session force-time curves exhibiting the lowest magnitude of variability were used for the comparisons. The session one eccentric knee extension mean repetition interval time was 3.27 s (*SD* = 2.22; CV = 68%) and the corresponding session two mean repetition interval time was 5.58 s (*SD* =.69; CV = 12%).

**Figure 3:**
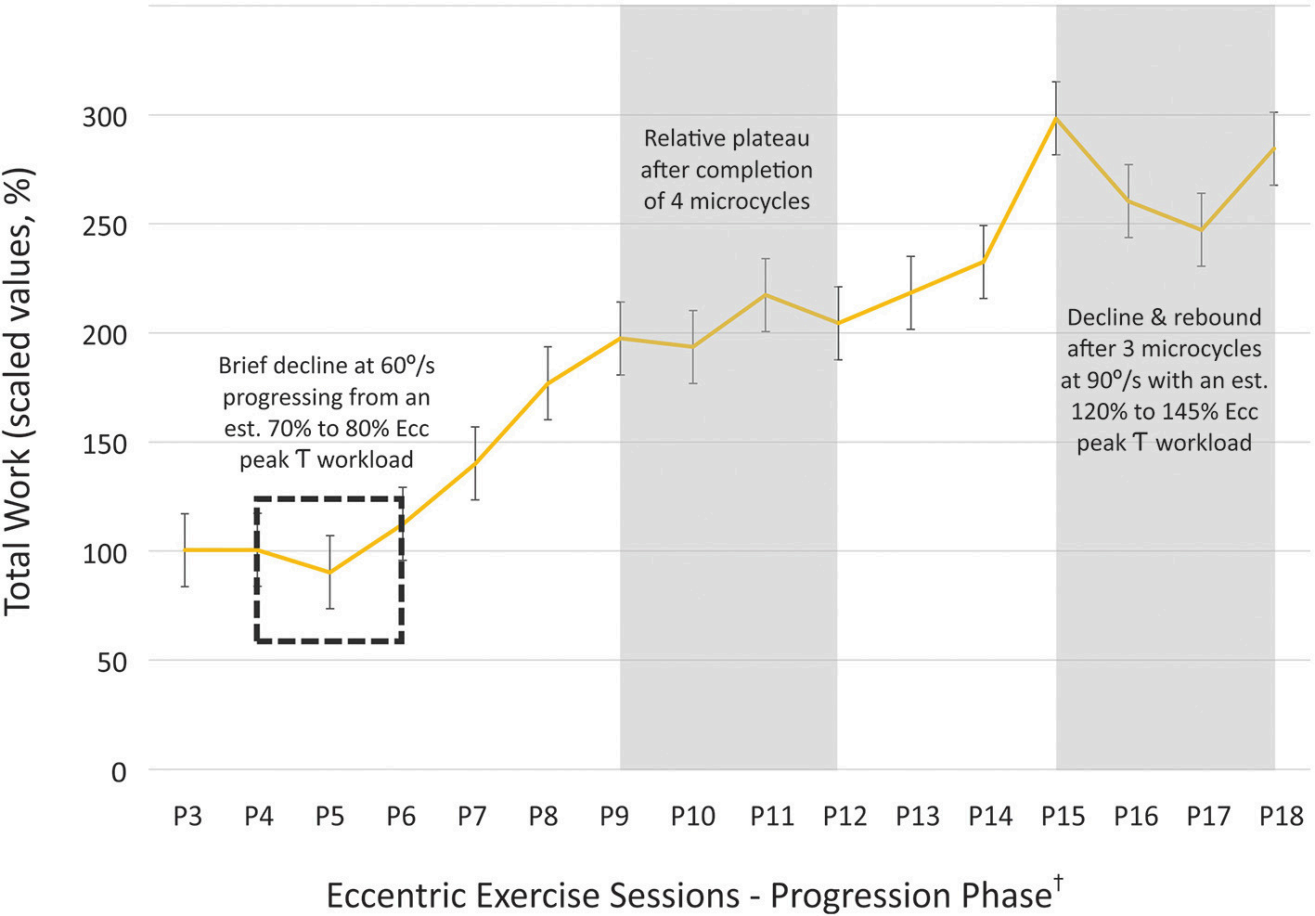
**Adjusted total work values attained during the progression phase of the strength training program**. Eccentric total work (60°/s) during the progression phase of the initial exercise program was indexed to the values attained at the start of the 5% workload increments during the fifth microcycle, and proportional change in the total work values was graphed until the end of the exercise program (^†^progression phase sessions 3 through 18; i.e., P3–P18). The total work increased from the index value of 100% to the final value of 285% at the end of the eccentric strength training regimen, and isolated periods of diminished exercise performance were identified as potential points in the exercise program suitable for an active recovery period. (τ, torque; error bars = *SE*, standard error).

Aggregate data was used to better understand the change in eccentric exercise capacity over time. The total eccentric work delta values for the 60° s^−1^ condition were indexed to the performance data attained during the beginning of the progression phase. The total work increased from the index value of 100% to the final value of 285% at the end of the eccentric strength training regimen (Figure 3). The slope of the progressive phase total work values was 13.4 (*SD* = 11.6; range = −5.5–29.6). The first plateau noted in the eccentric total work values occurred at the start of the eighth microcycle (slope = 4.48–6.18), and the only negative slope (−5.45) was detected at the start of eleventh microcycle.

**Figure 4:**
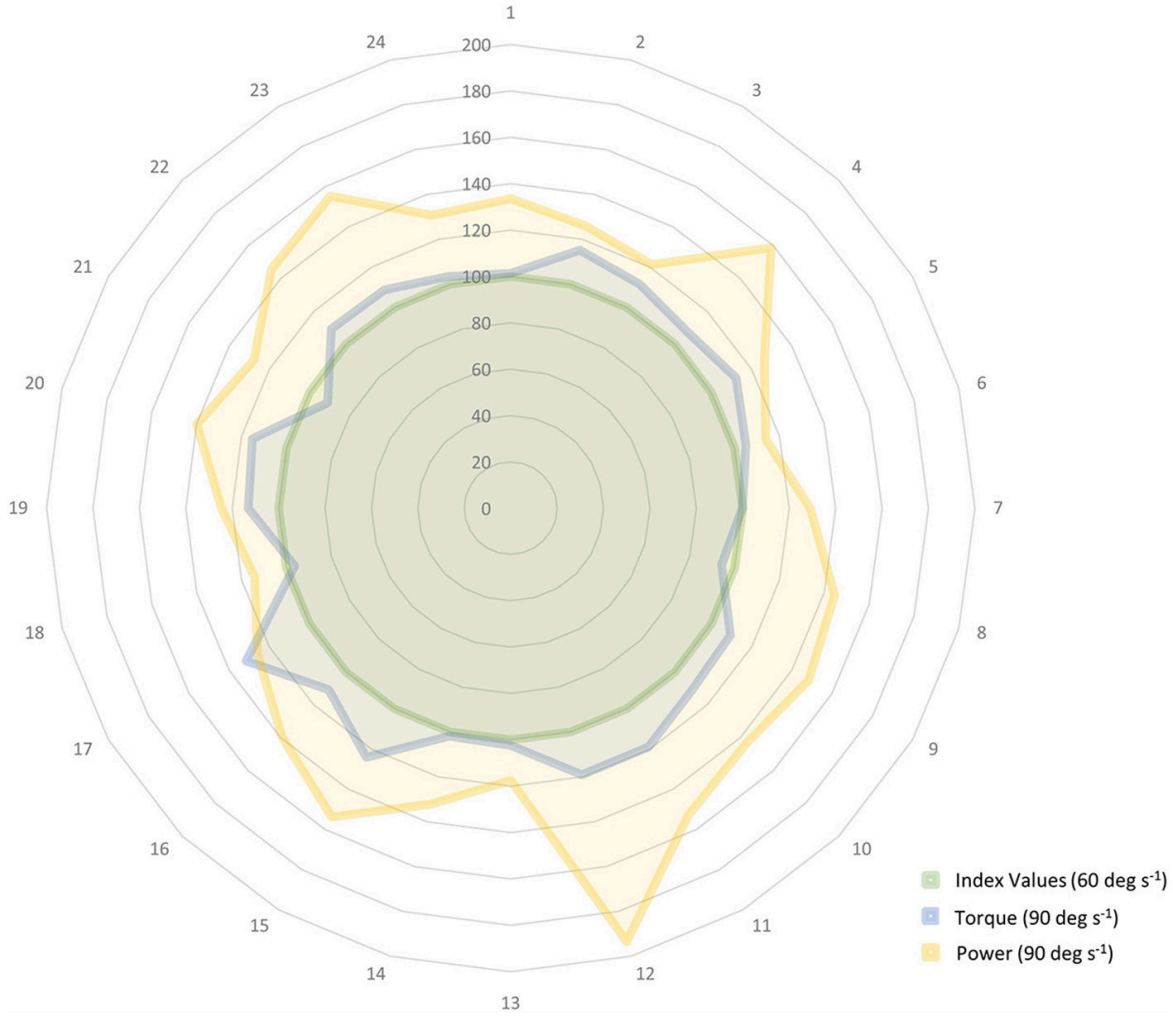
**Relative change in eccentric peak torque and mean power while using a fixed workload target during 60 and 90° s^−1^ isokinetic knee extension exercise**. The velocity-dependent behavior in eccentric torque and power generation are depicted in the radar graph. Torque and power data are indexed to the values obtained during the 60° s^−1^ condition (e.g., 100%, as shown in the innermost circle and centerline values) and the participant identification numbers are listed along the periphery of the circle. The participants used the same workload targets during exercise at both isokinetic speeds. The data attained during the 90° s^−1^ condition show that the attained peak torque values were comparable to those recorded during the 60° s^−1^ condition (Δ 7%). However, a greater proportional difference in mean power generation was noted in the 90° s^−1^ condition in comparison to the 60° s^−1^ condition (Δ 25%) within the sample. (deg, degrees; s, seconds).

The velocity-dependent behavior in eccentric torque and power generation within the sample was also explored. During the comparison of eccentric peak torque at 60 vs. 90° s^−1^ during the eighth microcycle, the former was 166.8 N m (*SD* = 78.1 N m) and the latter was 179.9 N m (*SD* = 80.2 N m). Regarding a similar comparison for mean power generation, these values were 78.3 W (*SD* = 47.6 W) at 60° s^−1^ and 103.9 W (*SD* = 56.9 W) at 90° s^−1^ (*p* < 0.0001; Figure 4).

## Discussion

The periodization model presented in this study for eccentric exercise familiarization and workload progression was feasible and safe to utilize within an outpatient rehabilitation setting based on our preliminary results. The implementation of this regimen served to highlight important considerations concerning the use of accommodating resistance devices for the purpose of eccentric strength training, and the use of this approach in clinical populations.

### The eccentric exercise prescription and the initial mesocycle

Exercise “intensity” is a term frequently used to denote the exercise workload as a proportion of the one-repetition maximum (1RM). However, exercise intensity is also impacted by intra-session rest periods and other factors that influence the subjective and objective effort needed to complete a training session. The overall “load” experienced by an athlete or a patient is the sum total of the work performed via the conditioning sessions along with the demands of skills-based training and additional physical activities (Stone et al., 1981; Lorenz and Morrison, 2015; Hoover et al., 2016). Nevertheless, the primary component of exercise intensity within the context of the exercise prescription is the relative intensity or assigned “workload” (expressed as a percentage of 1RM). Special attention should be afforded to patients with orthopedic conditions with significant joint pathology or neurological disorders with sequalae that include excessive fatigue when considering workload assignment and familiarization to the eccentric training stimulus.

#### Eccentric workload assignment for patient populations

A measured approach should be used in establishing the initial eccentric exercise prescription in rehabilitation settings. True 1RM testing is widely recognized as a critical task in the determination of the targeted workload. However, there are instances where caution or relative contraindications may prevent 1RM testing in older adults and those with chronic conditions. Testing protocols and predictive equations for the 1RM value by Brzycki and others have been successfully used for patient populations in order to circumvent the challenge of true 1RM testing (McNair et al., 2011). The issue of 1RM testing is further complicated during the assessment for active muscle lengthening in people that are new to resistance exercise involving eccentric muscle actions. While pre-intervention eccentric 1RM values have been obtained in previous studies involving relatively healthy adults (Roig et al., 2008), there are instances where the relative disease severity may rule out this approach (American College of Sports Medicine, 2014). Additionally, initial testing modes involving maximal eccentric strength testing and exercise may cause excessive muscle damage and delayed onset muscle soreness (DOMS) that may adversely affect participant adherence and could further impair those with pre-existing physical limitations (Parr et al., 2009). The use of estimated eccentric 1RM values have been proposed in lieu of fully validated eccentric predictive 1RM equations (Harris-Love, 2005; Hernandez et al., 2015). Estimated eccentric peak torque values have been derived from the estimated eccentric/concentric peak torque differential reported by other investigators (Dudley et al., 1991). Peak eccentric force and torque estimates vary widely based on the method of assessment and the muscle groups tested, and may be ~20–100% higher than concentric values (Enoka, 1996; Hortobagyi et al., 2001; Kelly et al., 2015). In addition, the applied use of adjusted peak eccentric torque estimates ranging from 35 to 40% have been explored in patient populations (Harris-Love, 2005; Hernandez et al., 2015). Higher cofactors could be considered for use in other patient populations based on their exercise tolerance, joint integrity associated with the agonist/antagonist muscle groups, and general training goals. Underestimates of eccentric peak torque are possible when using cofactors below 1.5, but this may be an appropriate constraint for rehabilitation interventions. Also, the exercise workload ultimately rises to the ability of the individual patient during the iterative progression phase of the macrocycle. A comparison of cofactors used to determine peak eccentric torque estimates was beyond the scope of this study. However, the participants exhibited a decline in total work upon transitioning from a 70 to 80% of the estimated eccentric peak torque (Figure 3) early in the progressive phase of training. This suggests that use of a higher cofactor for the peak eccentric torque estimates may have resulted in workload targets too difficult to attain for the study participants. Nevertheless, additional work remains to be done to develop and cross-validate predictive equations for eccentric 1RM values in various patient populations and in samples that properly account for age and gender effects (Kellis et al., 2000).

#### “first do no harm”

Previous investigators have shown that prior muscle action history influences subsequent physiological responses to eccentric loading (McHugh et al., 1999; Nosaka et al., 2001; Margaritelis et al., 2015). Adaptations involving the skeletal muscle ultrastructure and the ability to withstand oxidative stress may occur in response to submaximal eccentric loading (Lima and Denadai, 2015; Deyhle et al., 2016). In addition, preliminary evidence suggests that this protective effect is associated with benign or modestly elevated inflammatory activity, rather than a diminished inflammatory response, and may be an adaptation to aid the recruitment of immune cells (Deyhle et al., 2016). This view is consistent with the observation that the post-exercise presence of macrophages may aid the recovery of skeletal muscle following eccentric exercise bouts (Tidball and Wehling-Henricks, 2007). Moreover, DOMS may be disassociated from the post-exercise inflammatory response since increased chemokine levels accompany the successful inducement of the “repeated bout effect” (Deyhle et al., 2016). The repeated bout effect represents the ability of skeletal muscle to resist exercise-induced damage following a preceding exposure to the exercise stimulus. Intentional use of the repeated bout effect is now an accepted precept of eccentric exercise programming (Flann et al., 2011; Gluchowski et al., 2015; Margaritelis et al., 2015) and it remains a core element of the applied use of eccentric muscle actions as a form of therapeutic exercise.

The magnitude of the repeated bout effect may be a function of the time course from the exposure stimulus to the exercise stimulus, range of motion used during the initial exposure stimulus, volume of the exposure stimulus, age of the participant, and muscle action type employed (Lavender and Nosaka, 2006; Lima and Denadai, 2015). The ideal period between the exposure stimulus and progressively higher levels of loading may be within 2–4 days when considering factors such as DOMS and markers of muscle damage such as creatine kinase activity (Lima and Denadai, 2015). However, extended periods of the repeated bout effect, based on the criterion of force production and other measures of muscle status, have been conferred by higher levels of eccentric exposure stimuli (Nosaka et al., 2001). An initial exposure to eccentric exercise via high workloads is problematic for those undergoing rehabilitation. Therefore, submaximal eccentric or isometric muscle actions with incremental loading over multiple sessions may be used to prepare patients for an eccentric PRE regimen. In this study, we reported that the participants had fairly stable VAS values for musculoskeletal pain (VAS values were 2.6 ± 2.9 at the highest workload targets during Week 12). Our findings are in agreement with other investigators (LaStayo et al., 2007) regarding the use of gradual eccentric loading to minimize DOMS in older adults. In the proposed periodization model, we have formalized an approach to the volume and time course of the submaximal eccentric exercise bouts (within the first mesocycle) using an isokinetic mode of strength training. This structured approach to the first mesocycle may aid task performance and facilitate the acquisition of protective adaptations with minimal deleterious effects.

#### Beyond the repeated bout effect

The initial exposure to eccentric muscle actions has value beyond the repeated bout effect. The proposed periodization macrocycle includes a distinct phase for gaining familiarization with eccentric exercise. This early phase of training includes important components of motor learning related to the mode of exercise and the control of movements involving active muscle lengthening. In addition, the participants begin to attempt incrementally higher workloads as they transition from the familiarization phase to the acclimatization phase. Distinct differences exist between the neural control of eccentric and concentric muscle actions. In untrained individuals, full activation of muscle is difficult to achieve during voluntary eccentric muscle actions in comparison to concentric muscle actions (Amiridis et al., 1996; Kellis and Baltzopoulos, 1998; Aagaard et al., 2000). This may be due to spinal (Pinniger et al., 2000) and supraspinal (Gruber et al., 2009) mechanisms that constrain motor unit discharge rates to protect against potential muscle damage caused by high eccentric force levels. Importantly, muscle action-specific differences in motor unit excitability may be influenced by the activation of different cortical areas during eccentric exercise in comparison to concentric exercise (Kwon and Park, 2011). Also, despite the relative retention of eccentric muscle strength with advancing age (Power et al., 2012), cortical activation patterns for movements involving eccentric muscle actions may become impaired in older adults (Yao et al., 2014). These observed neurological changes may contribute to age-related movement deficits with tasks that involve significant contributions from actively lengthening muscle groups (Chung-Hoon et al., 2016).

The preliminary observations gleaned from the participants in this study suggest that a substantial decrease in motor performance variability occurs between the start of the familiarization phase to the start of the acclimatization phase. This interpretation is based on the gradual normalization of the torque-time curve features during the early weeks of training. The exemplar data shown in the Figure 1 shows the variability typically seen in the torque-time curves that result from submaximal eccentric muscle activity during a basic isokinetic knee extension and flexion task (CV = 68% during the 2nd set of session 1 vs. 12% during the 3rd set of session 2). The ability to produce eccentric torque-time curves with minimal variability differs across individuals, but is generally attained within the first one to three microcycles of the eccentric exercise program. This learning process may also be facilitated by the low target eccentric workload during the early phases of the regimen, and the knowledge of performance gleaned from visual feedback provided by the dynamometry system computer monitor.

### The eccentric power-velocity paradox

**Figure 5:**
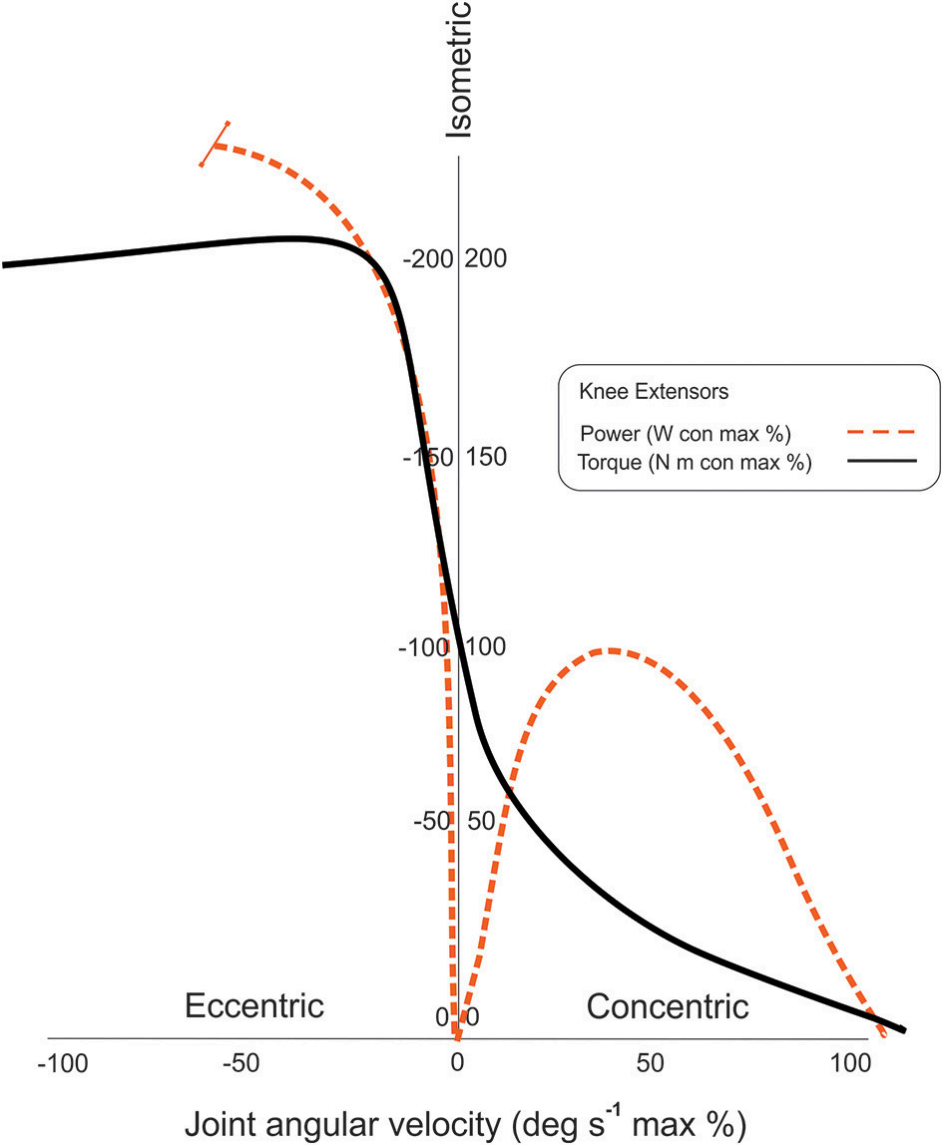
**Velocity-dependency of eccentric peak torque and mean power**. The power-velocity relationship for eccentric muscle actions differs greatly in comparison with concentric muscle actions as shown in the idealized graph. Peak concentric power is attained at intermediate velocities and peak torque levels, whereas, peak eccentric power rises precipitously with increasing velocities and fairly stable high peak torque levels. (The indeterminate physiologic decline in power at the highest velocities is denoted by the terminal bar on the dashed trajectory line.) (con, concentric; max, maximum).

The power-velocity relationship for eccentric muscle actions differs greatly in comparison with concentric muscle actions, and has important implications for eccentric exercise programming (Figure 5). Tom McMahon and Jason Harry's memorable description of “the dark side of the force-velocity curve” aptly describes the altered behavior of contractile tissue during eccentric muscle actions (Lindstedt et al., 2001). This depiction largely captures the observation of sustained high eccentric peak force generation with increasing movement velocity in contrast with concentric muscle actions. However, McMahon and Harry's insight also extends to the unique attributes of power generation during eccentric muscle actions. Unlike concentric muscle actions, power appears to increase at very high velocities without an appreciable loss of peak force when active muscles lengthen. Peak force immediately decreases upon transitioning from isometric to concentric muscle actions, and the parabolic curve in muscle power predictably rises as velocity increases, but eventually declines at relatively high velocities (Figure 5). This observed physiologic decline in concentric muscle power has been attributed to the time course required for maximal concentric muscle activation and suboptimal actin/myosin cross-bridge mechanics at high velocities (Hutchins et al., 1998; Cramer et al., 2002; Demura and Yamaji, 2006; Power et al., 2015). However, in considering the eccentric power-velocity paradox, eccentric muscle actions may bias skeletal muscle toward maximal power generation at high velocities. This unique feature of eccentric muscle actions may be attributable to the viscoelastic properties of the muscle-tendon complex, the inherent properties of sarcomeric cytoskeleton proteins such as titin and myomesin that may serve to aid energy conservation, and active force enhancement via the hypothesized Ca^2+^ dependent binding in the N2A region of titin (Agarkova and Perriard, 2005; Gautel and Djinovic-Carugo, 2016; Nishikawa, 2016).

The eccentric power-velocity relationship was assessed using the study participants' knee extension exercise data. The progression phase of the initial macrocycle included a gradual increase in the angular velocity of the eccentric exercise with the isokinetic parameters increasing from 60 to 90° s^−1^ over the course of the final mesocycle (Table 1). While peak torque was expected to remain stable between the two exercise conditions, it did exhibit a modest increase of 7%. Studies conducted by other investigators have shown both unchanging or increasing peak torque values secondary to higher movement velocities, and training status may be a potential factor in the variation of force or torque levels (Hortobágyi and Katch, 1990; Cramer et al., 2002; Power et al., 2015). Velocity-dependent muscle performance may also vary for different muscle groups based on the joint type and mode of testing (Mayer et al., 1994). Given the increased propensity of subjects to exceed the visual torque targets at high velocities, the observed increase in peak torque in this study may be due to the limitations of the test conditions rather than a significant deviation of the expected eccentric force-velocity relationship. Regarding the power-velocity relationship, the mean power attained by the participants was 25% higher at 90° s^−1^ in comparison to the 60° s^−1^ condition (Figure 4). This increase in power exceeds what could be attributed to the low magnitude of difference in peak torque at 90° s^−1^ and appears to be similar to the findings from other investigators (Wu et al., 1997; Cramer et al., 2002). While this study was conducted under controlled conditions, the present findings were derived from exercise data, so order effects may influence the interpretation of the findings. Nevertheless, the reported power data appear to be consistent with the expected eccentric power-velocity relationship.

The initial eccentric exercise macrocycle included progressive increases in workload and movement velocity to obtain post-exercise adaptations in muscle strength and power. The mean age of the study participants was nearly 62 years, and age-related decreases in both muscle strength and power have been implicated in the increased incidence of mobility limitations and falls in older adults (Clynes et al., 2015). Higher velocity strengthening regimens—using both concentric and eccentric muscle actions—have been proposed as an activity-based strategy to counteract these adverse changes in muscle performance via exercise specificity (Caserotti et al., 2008; Power et al., 2015). However, unlike with concentric muscle actions, increased movement velocity during eccentric muscle actions may significantly affect exercise intensity even when program variables such as workload and repetitions remain unchanged. Practitioners should be aware that peak eccentric muscle torque or force may remain high during activities designed to maximize the production of peak eccentric power.

### An eccentric exercise macrocycle for accommodating resistance: the progression phase

The lower levels of anaerobic fatigue noted with repeated eccentric muscle actions in comparison to concentric muscle actions (Enoka, 1996; Ratamess et al., 2016), coupled with the use of accommodating resistance devices, invites unique challenges regarding workload progression and exercise stoppage criteria for eccentric strengthening regimens.

#### Accommodating resistance and the decision algorithm for workload adjustments

Isoinertial exercise using free weights or machines that utilize stack weights remain the dominant mode of strength training (Cotterman et al., 2005). However, many rehabilitation facilities and sports medicine clinics feature instrumented variable resistance devices for the assessment of motor performance and as a primary or adjunctive method of strength training for patients. Fundamental differences exist between isoinertial and variable resistance exercise (Avrillon et al., 2016). Variable resistance exercise performed on devices such as cam-based machines provide altered resistance levels throughout the range of motion. The application of variable resistance is designed in a manner presumed to optimize the skeletal muscle length-tension relationship (Frost et al., 2010). Isokinetic exercise avoids the limitations of cam designs that may not be ideal for a given participant's body proportions, and instead employs a strategy of constraining motion via selected peak angular velocities (Hislop and Perrine, 1967). Forms of variable resistance exercise such as isokinetics and ergometry provide accommodating resistance and thus allow motion to continue unabated even if participants are unable to meet target workload levels. During a fatiguing bout of isoinertial exercise, the participant will reach repetition failure (or produce a partial repetition) and cease the exercise activity. In contrast, fatiguing bouts of isokinetic or ergometry exercise are typically characterized by a decline in peak torque or watts (without a loss of joint excursion) over the course of a predefined number of repetitions or a fixed training period. As a result, identifying repetition “failure,” missed workload targets, or an inability to meet volume or total work goals during isokinetic or ergometry exercise requires readily available data and an algorithm to make workload adjustments.

Exercise regimens involving the use of accommodating resistance may require the acquisition and interpretation of intra-session anaerobic fatigue data to calculate appropriate workload adjustments. Visual torque targets were used to aid the decision algorithm featured in this study for workload adjustments as shown in Figure 2. Computer-assisted isokinetic dynamometry allows for the real-time assessment of torque-time curves and participant visual feedback concerning the prescribed workload. The isokinetic dynamometer used in this study allowed for the entry of torque limits as a safety precaution during the exercise sessions. Individuals typically cannot consistently attain their intra-session workload goal if the safety torque limit value is too close to the visual torque target value. Safety torque limits may be established up to 50 N m above the prescribed workload to ensure that the device safety mechanism does not unnecessarily impede exercise performance within acceptable workload parameters. Adjustments in the target workload and movement velocity may also be used to aid patient safety and optimize the load progression within each microcycle of a periodized eccentric training regimen.

Finally, incorporating a decision algorithm for workload adjustments and exercise stoppage based on reported pain is a critical component of eccentric exercise programming involving patient populations. The exercise stoppage criterion was a VAS pain score of >8 given that adults with musculoskeletal impairments can have relatively high baseline levels of pre-activity pain that do not preclude exercise participation. Nonetheless, a large proportional increase of exercise-related pain in patients with low baseline VAS pain ratings may merit exercise cessation even if their absolute pain level is below the stoppage criterion. Therefore, the implementation of strength training programs for the rehabilitation of patients with chronic conditions should continue to involve the consultation of the interdisciplinary health professional team.

#### Assessing the progression phase of the macrocycle

Notably, the inclusion of the familiarization, acclimatization, and progression phases within the macrocycle suggest that the proposed periodization model is designed for individuals that are new to eccentric exercise. People with minimal training experience may exhibit relatively fast adaptations to strength exercise in comparison to those with extensive training experience (Mangine et al., 2015), and the workload is often progressed following each exercise bout or microcycle as needed (Beurskens et al., 2015; Unhjem et al., 2015). The choice to use a relatively low cofactor to calculate the estimated eccentric peak torque used for workload assignment, coupled with the general fatigue resistance exhibited by actively lengthening muscles, informed the decision to use a common linear PRE progression in this study. While there is some evidence to support the use of non-linear program designs for optimal strength gains (Fleck, 2011; Miranda et al., 2011), the findings are equivocal and the use of complex periodization schemes confer little advantage to untrained individuals with general strengthening goals (Lorenz et al., 2010). Nevertheless, periodized strength training regimens may be more effective than non-periodized programs (Rhea and Alderman, 2004), and formal loading and recovery phases are key elements within the structure of the macrocycle (Lorenz and Morrison, 2015). Optimal recovery phases for eccentric training regimens are ill defined. It is not fully understood if the recovery phases for eccentric strength training differ from conventional PRE programs given the high torque output and decreased anaerobic fatigue associated with eccentric muscle actions.

Aggregate data from the study participants were used to examine the progression phase of the eccentric training regimen in order to determine when the cyclic use of decreased loading should be integrated into the mesocycle. The eccentric exercise performance of the participants was assessed by an examination of the work-time line graph obtained during the progression phase of the regimen. It was presumed that relatively level slopes were an indicator of a training plateau, and that negative slopes denoted periods of decreased exercise capacity. In reviewing the progressive phase of the program, consecutive slope values <10 calculated across a span of microcycles occurred only at the midpoint and endpoint of the mesocycle. Slope values (4.48–6.18) indicating a relative plateau in the attained total work occurred following the completion of four microcycles with visual torque targets set at >70% of the estimated eccentric peak torque (Figure 3). The need for a recovery period within a mesocycle following 3–5 weeks of training is not uncommon for conventional PRE regimens (Lorenz et al., 2010), and may also be suitable for eccentric PRE regimens with exercise intensity and volume levels similar to those used in this study. The only negative slope value detected within the progressive phase of the program was during the last two microcycles featuring the highest visual torque targets (nearly 150% of the initial estimated eccentric peak torque value). The instance of the relative decline in attained total work may have been influenced by the high workload targets, the higher estimated eccentric peak power associated with faster angular velocities, and the potential need for a recovery period at the midpoint of the mesocycle. High velocity movements across all exercise sets was a feature of the last three microcycles of the progression phase. The total work attained predictably increased during the initial eccentric training sessions that included all exercises performed at 90° s^−1^ (points P13 to P15 on Figure 3), so the introduction of increased movement velocity alone does not explain the subsequent incident decline in exercise capacity. However, the collective increase in exercise demands related to peak power generation and progressively rising workload targets can certainly contribute to blunted exercise adaptations or diminished performance over an extended period of time. Although a rebound from the decline in total work is noted in the aggregate data (points P17 to P18 on Figure 3), the pattern of the work-time line graph shows that the decline in total work occurs four microcycles after the start of the preceding exercise performance plateau. This observation indicates that the use of recovery periods after four microcycles of progressive workload and movement velocity increases may benefit eccentric exercise programs similar to the one used in this study. The information obtained from the progression phase work-time data was used to revise the proposed eccentric exercise periodization model as shown in Table 2.

**Table 2:** **Revised Eccentric Training Macrocycle**.

**MACROCYCLE–REVISED**													
***Familiarization***	***Acclimatization***		***Progression***										
**Mesocycle1**			**Mesocycle 2A**						**Mesocycle 2B**				
**Micro 1**	**Micro 2**	**Micro 3**	**Micro 4**	**Micro 5**	**Micro 6**	**Micro 7**	**Micro 8**	**Micro 9**	**Micro 10**	**Micro 11**	**Micro 12**	**Micro 13**	**Micro 14**
**Low target workload**			**Initial training workload**	**Progressive increase in target workload and power generation**				**Active recovery**	**Progressive increase in target workload and power generation**				**Active recovery**
45° s^−1^, 3 × 10	60° s^−1^, 3 × 10		60° s^−1^, 3 × 10	60° s^−1^, 4 × 10	60° s^−1^, 3 × 1; 90° s^−1^, 1 × 10	60° s^−1^, 2 × 1; 90° s^−1^, 2 × 10	60° s^−1^, 1 × 1; 90° s^−1^, 3 × 10	–	60° s^−1^, 4 × 10	60° s^−1^, 3 × 1; 90° s^−1^, 1 × 10	60° s^−1^, 2 × 1; 90° s^−1^, 2 × 10	60° s^−1^, 1 × 1; 90° s^−1^, 3 × 10	–
**TARGET ECCENTRIC WORKLOAD (% PEAK TORQUE) AND PROGRESSION**													
40–50%	50%	60%	70%	±5%	±5%	±5%	±5%	–	±5%	±5%	±5%	±5%	–

*Micro, microcycle; deg, degrees; s, second*.

*The workload adjustment algorithm may be applied after each session from microcycle 5 to microcycle 14 (excluding active recovery periods)*.

*The featured periodized strength training program is proposed as an approach to introduce isokinetic eccentric training in outpatient rehabilitation settings. The program revisions were informed by the participant exercise data featured in this study. The findings suggested that the use of active recovery periods following four consecutive microcycles (with conditional workload adjustments) would improve the structure of the program. Also, in an effort to avoid extended plateaus or decreased performance, use of non-linear cycling of the movement velocity was incorporated to better manage exercise intensity toward the end of the progression phase microcycles*.

### Implications and limitations of the proposed eccentric exercise periodization model

The workloads used at the outset of the progression phase (70% of the estimated eccentric peak torque) are consistent with the recommendations of the ACSM, but it should be noted that investigators have recently cited the efficacy of low workload/high volume in trained and untrained adults for increases in hypertrophy and strength (American College of Sports Medicine, 2014; Morton et al., 2016). Use of alternate exercise workload and volume programming may require mesocycle phases and recovery periods that differ from the findings of this report. These elements of the eccentric exercise regimen would also be impacted by the addition of other skills-based sports or rehabilitation activities since these physical demands are typically considered within the structure of the periodized program. The eccentric exercise regimen described in this study included the structured progression of both the workload and the movement velocity. Therefore, the separate effect of these variables on the exercise performance and estimated recovery periods cannot be determined from the data provided. Also, the descriptive use of exemplar data was used to illustrate participant exercise performance during the familiarization phase of the eccentric exercise program in this Theory and Hypothesis report. These observations, and reports on general program efficacy, will require follow up in subsequent clinical studies.

The isokinetic dynamometry used in this work, like all modes of exercise, has advantages and disadvantages that may vary based on individual training goals and the selected patient population. While exercise machines have been subject to criticism for not sufficiently reflecting multi-planar movement, isokinetic strengthening has shown some carryover effects to functional activities (Ratamess et al., 2016). Isokinetic strength training offered a high degree of utility in this investigation since it provided visual feedback regarding exercise performance, torque limits to enhance eccentric exercise safety, and the controlled increase of workloads while using only eccentric muscle actions. The singular use of isokinetic dynamometry as exercise for the knee extensors and flexors was for the purposes of this study, and does not reflect a comprehensive rehabilitation plan of care. In addition, a potential limitation of this work is that the range of angular velocities used for the eccentric exercise differed from the standard speeds used by our laboratory for strength assessment. Also, the contraindications for conventional strength training and eccentric exercise using external loads are critical to consider when providing the exercise prescription, and are beyond the scope of this report.

Comparing the implemented periodized eccentric strengthening regimen to other approaches to periodization was not an aim of this study. It is also important to emphasize that key differences exist among forms of eccentric strength training. Strength training regimens involving eccentric muscle actions should be conceptualized as two distinct types of exercise based on the Lindstedt model of a spring in series with a damper: activities that involve maximal acceleration and the potential recovery of elastic recoil energy, and activities which are largely characterized by net forces that result in deceleration and the absorption of elastic recoil energy (Lindstedt et al., 2001). The periodized eccentric strengthening program presented in this report involved participants eliciting eccentric torque in an effort to decelerate the motion generated by the dynamometer. Additional approaches have been considered regarding the structure and progression scheme for exercise involving eccentric muscle actions and rapid force development to aid the latter phases of physical rehabilitation (Davies et al., 2015). Lastly, the findings of this report are limited by the participant sample and different conclusions may be reached in exercise groups featuring people with different comorbidities.

## Conclusions

The eccentric training periodization model introduced in this report includes allowances for patient safety and motor learning during the early phases of the macrocycle. In addition, the challenge of detecting and monitoring missed workload targets when using accommodating resistance exercise was highlighted in this work. A criterion-based method was presented to manage workload adjustments through the assessment of intra-session anaerobic fatigue. Eccentric exercise performed at higher velocities resulted in increased power generation without an abatement of peak torque in the study participants. This characteristic of the eccentric power-velocity curve may be manipulated as a training variable to modify exercise intensity or optimize exercise specificity. The anaerobic fatigue rate and power-velocity relationship for eccentric muscle actions differs greatly in comparison with concentric muscle actions. Nevertheless, the need for exercise recovery periods relative to training intensity for eccentric PRE may be similar to the recommendations for conventional PRE programs. The periodized eccentric training model proposed in this report was informed by the progression phase work-time data. The model features recommended recovery periods after every four microcycles with incremental workload progressions (Table 2). Additional investigation is needed to determine the efficacy of the reported eccentric exercise program in older adults with chronic conditions. The continued development of periodization approaches based the eccentric exercise paradigm may lead to testable hypotheses concerning optimal progression algorithms, recovery phases, and target workloads for eccentric strength training used in the management of selected chronic conditions or the rehabilitation of athletic injuries.

## Author contributions

MH was responsible for the study design; HH, BS, MH, and TG performed the study procedures; MH, TG, BS, DP, and HH were responsible for data management and verification; MH and TG analyzed the data; MH prepared figures; MH, BS, TG, HH, DP, and BH collaborated on the data interpretation; MH, BS, TG, HH, DP, and BH drafted the manuscript MH, BS, TG, HH, DP, and BH edited and revised the manuscript; MH, BS, TG, HH, DP, and BH approved final draft.

### Conflict of interest statement

The authors declare that the research was conducted in the absence of any commercial or financial relationships that could be construed as a potential conflict of interest.
